# Dose–response associations between physical activity and sedentary time with functional disability in older adults with or without frailty: a prospective cohort study

**DOI:** 10.3389/fpubh.2024.1357618

**Published:** 2024-04-24

**Authors:** Satoshi Seino, Takumi Abe, Yu Nofuji, Toshiki Hata, Shoji Shinkai, Akihiko Kitamura, Yoshinori Fujiwara

**Affiliations:** ^1^Research Team for Social Participation and Healthy Aging, Tokyo Metropolitan Institute for Geriatrics and Gerontology, Itabashi, Tokyo, Japan; ^2^The Tokyo Metropolitan Support Center for Preventative Long-term and Frail Elderly Care, Tokyo Metropolitan Institute for Geriatrics and Gerontology, Itabashi, Tokyo, Japan; ^3^Department of Nutrition Sciences, Kagawa Nutrition University, Sakado, Saitama, Japan; ^4^Health Town Development Science Center, Yao City Public Health Center, Yao City, Osaka, Japan; ^5^Tokyo Metropolitan Institute for Geriatrics and Gerontology, Itabashi, Tokyo, Japan

**Keywords:** disability, frailty, physical activity, sedentary time, IPAQ, dose–response, older adults

## Abstract

**Purpose:**

Evidence regarding the dose–response curve shapes of physical activity (PA) and sedentary time (ST) in older adults with functional disability (FD) is extremely limited. Moreover, these associations may differ depending on with/without frailty. We examined the dose–response associations between moderate-to-vigorous PA (MVPA) and ST with FD among older adults with/without frailty.

**Methods:**

We included 7,480 initially nondisabled adults (3,795 men and 3,685 women) aged 65–84 years in Ota City, Tokyo, Japan. MVPA and ST were evaluated using the International Physical Activity Questionnaire-Short Form. FD was prospectively identified using a nationally unified database of the long-term care insurance system. Frailty was determined using Check-List 15, validated against Fried’s frailty criteria. Multivariate-adjusted hazard ratios (HRs) and 95% confidence intervals (CIs) of MVPA and ST for FD were calculated, and dose–response curves were examined using restricted cubic splines.

**Results:**

During 3.6 years of follow-up, 1,001 (13.4%) participants had FD. Among all participants, compared with no MVPA, the HRs for FD reduced linearly up to approximately 2000 metabolic equivalents (METs)■min/week of MVPA, and the lowest HR (HR: 0.61, 95% CI: 0.51–0.74) was reached at around 3,000–4,000 METs■min/week. Although the shape of this association was consistent regardless of with/without frailty, the magnitude of the association tended to be stronger in frail older adults than in non-frail older adults. Compared with those for the median (300 min/day) of ST, the HRs for FD increased linearly as ST reached approximately 600 min/day or more, independent of MVPA, with a maximum HR of 1.31 (95% CI: 1.01–1.71) for 1,080 min/day among all participants. This association was more pronounced among non-frail older adults but not statistically significant among frail older adults.

**Conclusion:**

Higher MVPA levels consistently reduced the incidence of FD regardless of frailty in a significant inverse nonlinear dose–response manner. A significant positive nonlinear dose–response association between ST and FD risk was identified among non-frail older adults but not among frail older adults. Increasing MVPA and reducing prolonged ST are important for preventing FD among non-frail older adults. However, reducing ST alone may be insufficient; increasing MVPA, even if by only small increments, is highly recommended for frail older adults.

## 1 Introduction

Healthy aging, which involves developing and maintaining the functional ability to enable well-being in older age (1), is becoming increasingly important with the aging of the global population (2). Functional ability encompasses various aspects of a person’s capabilities, including meeting basic needs, learning, decision-making, mobility, relationships, and societal contributions (1). In a rapidly aging society such as Japan, preventing functional disability (FD) among older adults must be addressed from the perspectives of individual well-being and social security costs.

Increased physical activity (PA) and decreased sedentary time (ST) help prevent FD, noncommunicable diseases, all-cause cardiovascular disease, and cancer mortality among older adults (3). The 2020 World Health Organization PA and Sedentary Behavior Guidelines Development Group mentioned the global PA agenda (e.g., the lack of evidence on the shape of the dose–response curve of PA and/or ST with health outcomes) (4). After this event, the results of several large-scale dose–response meta-analyses using cardiovascular diseases (5–7), cancer (5, 7), and all-cause mortality (7, 8) as outcomes have been reported. Recently, an umbrella review of dose–response analyses of PA with all-cause mortality in older adults was also reported (9).

However, there are still some issues that need to be addressed. First, evidence of the shape of the dose–response curve of PA and/or ST with incident FD among older adults remains extremely limited. To our knowledge, only one study each of PA (10) and ST (11) has examined the dose–response association with FD. Chen et al. (10) reported that accelerometer-assessed moderate-to-vigorous PA (MVPA), regardless of accumulation patterns, or light PA (LPA) in bouts of <10 min, was significantly associated with a lower risk of FD in a linear dose–response manner. They also reported that although a higher total ST was significantly associated with an increased risk of FD, this association was not independent of MVPA (11). Secondly, the shape of the dose–response curve for PA and/or ST with incident FD may differ between individuals with and without frailty, which is a geriatric syndrome associated with increased risks of FD and mortality (12). A previous study (13) indicated that among non-frail older adults, the daily step count at which the risk of mortality plateaued was approximately 5,000–7,000 steps per day, whereas in frail older adults, the daily step count showed an inverse association with mortality at approximately 5,000 steps or more per day. In frail older adults, higher PA and less ST may have a greater impact on reducing the risk of FD than in non-frail older adults. As the prevalence of frailty increases and the health status of older populations becomes more diverse with aging (1), it is important to examine the threshold of PA/ST at which FD risk is reduced or increased, as well as the potential benefits of high-volume PA for individuals with and without frailty. If increased MVPA and/or reduced ST consistently decrease the risk of FD in a dose–response manner, even among frail older adults, this knowledge would be valuable in addressing frailty in old age.

Therefore, our study aimed to investigate the dose–response relationship between MVPA and ST with incident FD among older adults, taking into account the presence or absence of frailty.

## 2 Materials and methods

### 2.1 Study participants

This prospective study utilized data from a community-wide intervention study aimed at preventing frailty, which was initiated in Ota City, Tokyo, Japan in 2016 (14, 15). A previous report (14) has detailed the study design and participant characteristics. Briefly, 15,500 residents aged 65–84 years who were not certified as needing long-term care insurance (LTCI) (i.e., FD) (16, 17) as of June 1, 2016, were randomly selected from all 18 administrative districts of Ota city, stratified by age group (65–74 and 75–84 years) and sex (14).

**Figure 1:**
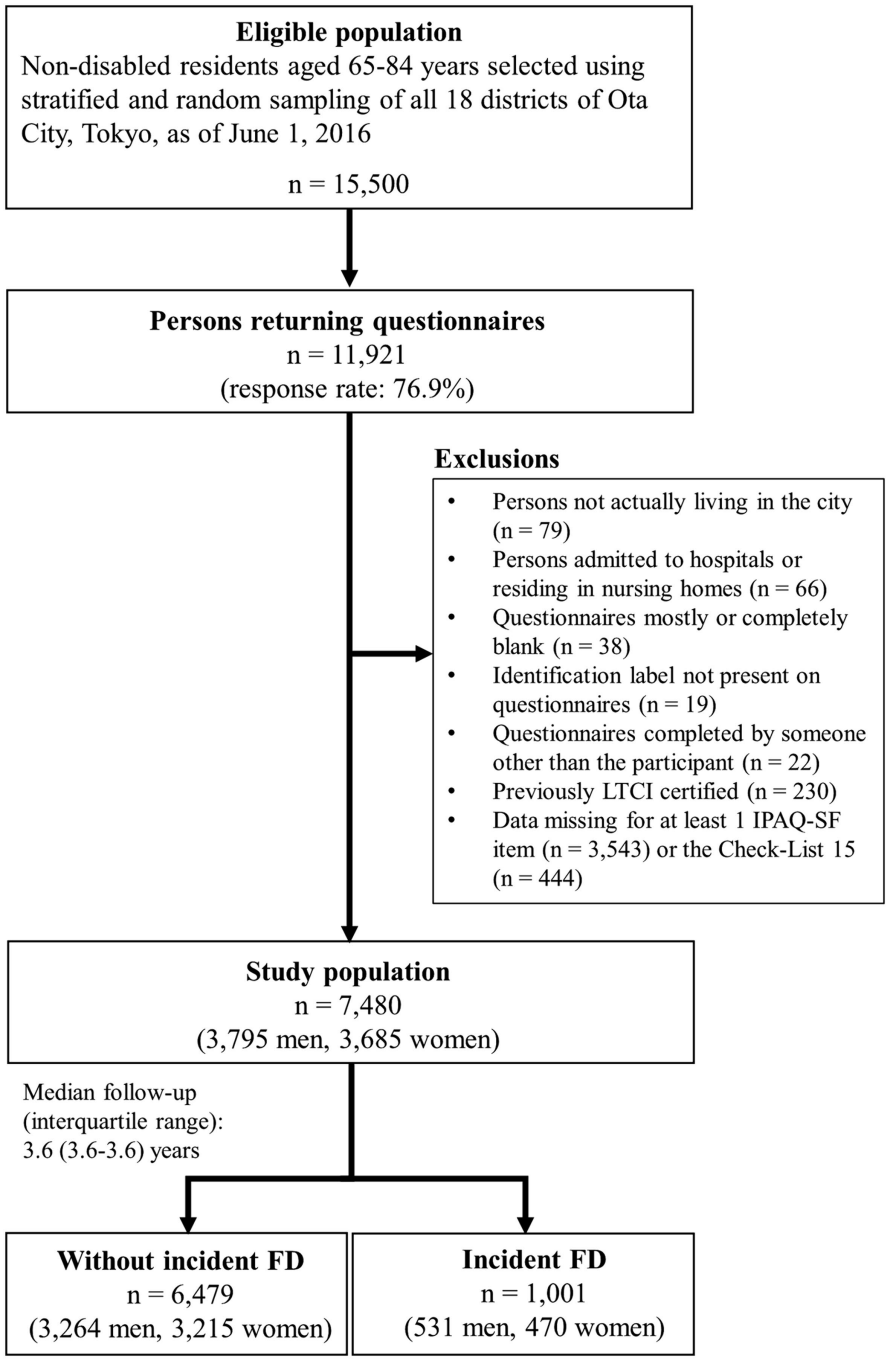
Flow diagram of the study participants. IPAQ-SF = International Physical Activity Questionnaire-Short Form.

A self-administered questionnaire survey was administered between July and August 2016. Of the 15,500 questionnaires distributed, 11,921 were returned (response rate: 76.9%). Finally, 7,480 questionnaires (3,795 men and 3,685 women) were included in this analysis according to the exclusion criteria shown in Figure 1.

The Ethical Committee of the Tokyo Metropolitan Institute for Geriatrics and Gerontology approved this study on June 1, 2016 (reference number: 8). All participants were informed that their participation in this study was voluntary, and they provided informed consent.

### 2.2 Measurements

#### 2.2.1 PA and ST

We used the Japanese version of the International Physical Activity Questionnaire-Short Form (IPAQ-SF) (18, 19) to evaluate the level of MVPA and ST. We assessed the time participants spent engaging in at least 10 min of vigorous-intensity physical activity (VPA), moderate-intensity physical activity (MPA), and walking during a typical week, as well as their usual weekday ST, excluding the time spent sleeping. Data cleaning and analysis were performed in compliance with the guidelines of the IPAQ-SF for data processing and analysis (20). According to the guidelines, PA intensities of 3.3, 4.0, and 8.0 metabolic equivalents (METs) were assigned for walking, MPA, and VPA, respectively (20). Responses at 0 min were treated as missing values in the ST analysis (21). Additionally, all instances of ST exceeding 1,080 min (18 h) per day were replaced with 1,080 min of ST (21). The total MVPA was classified as low (<600 METs■min/week; i.e., equivalent to <150 min/week of MPA), moderate (600–3,000 METs■min/week; i.e., equivalent to 150–750 min/week of MPA), and high (>3,000 METs■min/week; i.e., equivalent to >750 min/week of MPA), in compliance with previous studies (21, 22). The STs were classified as <180, 180–299, 300–479, and ≥ 480 min/day, based on previous studies (21, 23) and the median (300 min/day) in this study.

#### 2.2.2 Frailty

Frailty status was defined as a score of ≥4 on the Check-List 15 (original name: Kaigo-Yobo Checklist) (24, 25), a questionnaire validated against Fried’s frailty criteria (26). Check-List 15 is strongly correlated with the Frailty Index (27), a significant predictor of FD and/or mortality, and is compatible with the Frailty Index for predicting risk (25).

#### 2.2.3 FD

Participants’ FDs were identified using the mandatory database of the Japanese LTCI system, into which all adults aged 40 and above are enrolled. The system is responsible for providing formal care and support to Japanese adults aged 65 years and above with physical and mental disabilities (16, 17). The LTCI certification process is based on a multistep evaluation that adheres to nationally established standards (16). Briefly, upon the request of an older adult, his/her family, or caregiver, a trained local government official visits the home to assess the applicant’s long-term care needs using a nationally standardized questionnaire consisting of 74 items regarding current physical and mental status. The applicant’s attending physician or a physician designated by the local government prepares a written opinion (28, 29) according to a standardized physician manual for the LTCI. Next, a standardized computer-based system makes a first judgment based on this information. Ultimately, the final decision regarding certification is made by the Municipal Certification Committee, based on the results of the first judgment and special notes. The committee classifies care needs into seven levels (support level: 1–2; care level: 1–5; care level 5 indicates the most severe FD) (16).

FD was defined as the occurrence of long-term care needs at support level 1 or above (10), using the date of the LTCI application as the date of the FD incident. Since the novel coronavirus disease pandemic caused new LTCI application deferrals among older adults in this cohort (30), we set January 31, 2020, before the impact of the pandemic, as the endpoint.

#### 2.2.4 Covariates

We considered covariates based on previous studies that examined the relationship between PA, ST, and FD (10, 11, 31) in addition to socioeconomic status. The covariates were age, sex, district, living with others or alone, marital status (married, widowed, divorced, or never married), education (junior high school, high school, or junior college/vocational college/college/graduate school graduate), equivalent income (<2.0, 2.0–3.99, ≥4.0 million yen, or unknown) (32), body mass index (BMI) (<18.5, 18.5–24.9, or ≥ 25 kg/m^2^), self-rated medical conditions (hypertension, dyslipidemia, heart disease, stroke, diabetes mellitus, and cancer), alcohol drinking and tobacco smoking statuses (current, never, or former), lower back pain, and knee pain (presence or absence).

#### 2.2.5 Statistical analyses

Data were analyzed using Stata 18.0 (StataCorp, College Station, TX, USA). An α of 0.05 indicated statistical significance. The baseline characteristics of the study population were compared between those with and without incident FD using the unpaired t-test, Mann–Whitney U test, or chi-square test.

We used Cox proportional hazards models for our primary analysis with FD as the dependent variable and MVPA or ST categories as independent variables. The person-years of follow-up were calculated for each individual from July 1, 2016, until the occurrence of incident FD (the date of the LTCI application), migration from Ota City, or the end of the follow-up period (January 31, 2020), whichever came first. Three analytical models were constructed. Model 1 was adjusted for age and sex. Model 2 was additionally adjusted for district, living situation, marital status, educational attainment, equivalent income, BMI, hypertension, dyslipidemia, heart disease, stroke, diabetes mellitus, cancer, alcohol consumption, tobacco smoking status, lower back pain, knee pain, and frailty. Model 3 was further adjusted for either MVPA or ST according to the independent variables. To examine whether the results varied according to sex, we examined the statistical interaction for FD between the MVPA or ST categories and sex. In the primary analysis, missing values for covariates were categorized as “missing” and incorporated into the analytical model.

Furthermore, in Model 3, we analyzed the dose–response curves of MVPA and ST with incident FD using restricted cubic splines commands (i.e., “mkspline,” “levelsof,” and “xblc” in Stata). We employed the Akaike information criterion (AIC) to select a restricted cubic spline with three (10th, 50th, and 90th percentiles), four (5th, 35th, 65th, and 95th percentiles), or five (5th, 27.5th, 50th, 72.5th, and 95th percentiles) knots, and chose the model with the lowest AIC (33). We set the reference values of 0 METs■min/week or median ST (300 min/day) for each model (21).

We performed a stratified analysis for participants with/without frailty status using the same statistical methods except excluding frailty from the covariates to examine whether the dose–response association between MVPA and ST with FD differs depending on the presence or absence of frailty. The statistical interaction between MVPA or ST categories and frailty was also examined.

Two sensitivity analyses were conducted. First, to minimize selection bias, we used Cox proportional hazards models with multiple imputation by chained equations to impute missing values of covariates. We analyzed the 20 imputed datasets independently and combined the results for inference. Second, to reduce possible reverse causation, we conducted reanalyzes using the same statistical approaches after excluding individuals with incident FD during the first year of follow-up.

## 3 Results

Of the 7,480 people whose FD status could be confirmed during 3.6 years of follow-up (follow-up rate of 99.99%), 1,001 individuals (13.4%; 531 men and 470 women) had FD. Of them, 281 (28.1%; 161 men [30.3%] and 120 women [25.5%]) were diagnosed with FD during the first year. The incidence of FD per 1,000 person-years was 40.7.

Table 1 provides the baseline characteristics of the study population according to FD status. The medians (interquartile ranges) were 0 (0–75) min/week for VPA, 0 (0–120) min/week for MPA, 300 (120–600) min/week for walking time, 1,485 (594–3,336) METs■min/week for MVPA, and 300 (180–480) min/day for ST. Compared to people without incident FD, those with FD had significantly lower MVPA, educational attainment, equivalent income, prevalence of dyslipidemia, and both lower and higher BMIs. Additionally, they were significantly older, more likely to live alone, less likely to be married, and less likely to be current drinkers. They also had longer ST and a higher prevalence of hypertension, heart disease, stroke, diabetes mellitus, cancer, lower back pain, knee pain, and frailty. No significant group differences were observed in terms of sex or smoking status. The baseline characteristics of the study population with and without frailty according to FD status are shown in Supplementary Tables 1, 2. Supplementary Table 3 shows the baseline characteristics of the 7,480 participants included in the analysis and the 3,987 participants excluded from the analysis. The incidence rate of FD was significantly higher in people excluded from analysis (20.1%) than in those included in the analysis (13.4%).

**Table 1:** Baseline characteristics of the study population according to FD status.

	All		Without incident FD		Incident FD		*p*
	(*n* = 7,480)		(*n* = 6,479)		(*n* = 1,001)		
VPA (min/wk), median (interquartile range)	0	(0–75)	0	(0–90)	0	(0–20)	<0.001
MPA (min/wk), median (interquartile range)	0	(0–120)	0	(0–120)	0	(0–50)	<0.001
Walking time (min/wk), median (interquartile range)	300	(120–600)	300	(180–480)	210	(60–420)	<0.001
MVPA (METs·min/wk), median (interquartile range)	1,485	(594–3,336)	1,584	(678–3,465)	1,040	(297–2,708)	<0.001
Low (<600 METs·min/wk), *n* (%)	1921	(25.7)	1,536	(23.7)	385	(38.5)	
Moderate (600–3,000 METs·min/wk), *n* (%)	3,512	(47.0)	3,100	(47.9)	412	(41.2)	<0.001
High (>3,000 METs·min/wk), n (%)	2047	(27.4)	1843	(28.5)	204	(20.4)	
ST (min/day), median (interquartile range)	300	(180–480)	300	(180–480)	360	(210–590)	<0.001
<180, *n* (%)	1,306	(17.5)	1,160	(17.9)	146	(14.6)	<0.001
180–299, *n* (%)	1788	(23.9)	1,584	(24.5)	204	(20.4)	
300–479, *n* (%)	2070	(27.7)	1801	(27.8)	269	(26.9)	
≥480, *n* (%)	2,316	(31.0)	1934	(29.9)	382	(38.2)	
Age (years), mean (SD)	73.7	(5.5)	73.1	(5.3)	77.3	(4.9)	<0.001
Sex (men), *n* (%)	3,795	(50.7)	3,264	(50.4)	531	(53.1)	0.116
Living alone, *n* (%)	1,491	(19.9)	1,261	(19.5)	230	(23.0)	0.026
Marital status, *n* (%)							<0.001
Married	5,091	(68.1)	4,451	(68.7)	640	(63.9)	
Widowed or divorced	1733	(23.2)	1,447	(22.3)	286	(28.6)	
Never married	576	(7.7)	518	(8.0)	58	(5.8)	
Education, *n* (%)							<0.001
Junior high school graduation	1,562	(20.9)	1,290	(19.9)	272	(27.2)	
High school graduation	2,824	(37.8)	2,457	(37.9)	367	(36.7)	
Junior college/vocational college/ college/graduate school graduation	2,909	(38.9)	2,582	(39.9)	327	(32.7)	
Other/missing	185	(2.5)	150	(2.3)	35	(3.5)	
Equivalent income, *n* (%)							<0.001
<2.0 million yen	1,202	(16.1)	999	(15.4)	203	(20.3)	
2.0–3.99 million yen	2,633	(35.2)	2,232	(34.5)	401	(40.1)	
≥4.0 million yen	2,277	(30.4)	2020	(31.2)	257	(25.7)	
Unknown/missing	1,368	(18.3)	1,228	(19.0)	140	(14.0)	
BMI (kg/m^2^), mean (SD)	22.7	(3.1)	22.7	(3.1)	22.6	(3.5)	0.038
<18.5, *n* (%)	586	(7.8)	468	(7.2)	118	(11.8)	
18.5–24.9, *n* (%)	5,278	(70.6)	4,626	(71.4)	652	(65.1)	<0.001
≥25, *n* (%)	1,578	(21.1)	1,357	(20.9)	221	(22.1)	
Hypertension, *n* (%)	3,941	(52.7)	3,359	(51.8)	582	(58.1)	<0.001
Dyslipidemia, *n* (%)	3,125	(41.8)	2,745	(42.4)	380	(38.0)	<0.001
Heart disease, *n* (%)	1,652	(22.1)	1,354	(20.9)	298	(29.8)	<0.001
Stroke, *n* (%)	516	(6.9)	402	(6.2)	114	(11.4)	<0.001
Diabetes mellitus, *n* (%)	1,315	(17.6)	1,094	(16.9)	221	(22.1)	<0.001
Cancer, *n* (%)	1,198	(16.0)	974	(15.0)	224	(22.4)	<0.001
Alcohol drinking status (current), *n* (%)	4,288	(57.3)	3,798	(58.6)	490	(49.0)	<0.001
Smoking status (current), *n* (%)	958	(12.8)	831	(12.8)	127	(12.7)	0.288
Lower back pain, *n* (%)	2,799	(37.4)	2,334	(36.0)	465	(46.5)	<0.001
Knee pain, *n* (%)	2,244	(30.0)	1859	(28.7)	385	(38.5)	<0.001
Frailty, *n* (%)	1737	(23.2)	1,290	(19.9)	447	(44.7)	<0.001

BMI = body mass index; FD = functional disability; METs = metabolic equivalents; MPA = moderate physical activity; MVPA = moderate-to-vigorous physical activity; SD = standard deviation; ST = sedentary time; VPA = vigorous physical activity.

Table 2 shows multivariate-adjusted hazards ratio (HRs) and 95% confidence intervals (CIs) of MVPA and ST for incidence of FD. Among all participants, the moderate (HR: 0.75, 95% CI: 0.65–0.86) and high (HR: 0.68, 95% CI: 0.57–0.82) MVPA groups experienced significantly and gradually reduced incidence of FD compared to the low MVPA group, even in the Model 3 (*p* < 0.001 for trend). Although the statistical significance of the dose–response relationship between ST and FD became marginal when adjusted for frailty in Model 2 (*p* = 0.073), it completely disappeared when adjusted for MVPA in Model 3 (*p* = 0.149). The statistical significance of MVPA and ST for incidence of FD among those with/without frailty was similar to those for all participants, although the HR for FD in the high MVPA group was slightly attenuated among non-frail older adults. These results of associations of MVPA and ST with incidence of FD were did not vary by sex (*p* for interactions ≥0.122) and presence or absence of frailty (*p* for interactions ≥0.075). The results of the Cox proportional hazards model, in which missing covariates were compensated for by multiple imputation, were almost the same as those of the primary analysis (Supplementary Table 4). Moreover, the analysis results that excluded the incidence of FD during the first year of follow-up did not substantially differ from those of the primary analyses (Supplementary Table 5).

**Table 2:** Multivariate-adjusted HRs and 95% CIs of MVPA and ST for incident FD.

Variables	Number of events per participants	Incidence rate per 1,000 PY	Model 1			Model 2			Model 3		
			HR	(95% CI)	*p*	HR	(95% CI)	*p*	HR	(95% CI)	*p*
All participants (*n* = 7,480)											
MVPA											
Low (<600 METs·min/wk)	385/1921	64.2	1.00	(Ref.)		1.00	(Ref.)		1.00	(Ref.)	
Moderate (600–3,000 METs·min/wk)	412/3512	35.1	0.59	(0.51–0.68)	<0.001	0.74	(0.64–0.85)	<0.001	0.75	(0.65–0.86)	<0.001
High (>3,000 METs·min/wk)	204/2047	29.7	0.52	(0.44–0.62)	<0.001	0.68	(0.57–0.81)	<0.001	0.68	(0.57–0.82)	<0.001
	1001/7480	40.7	Trend		<0.001	Trend		<0.001	<0.001		<0.001
ST											
<180 min/day	146/1306	33.8	1.00	(Ref.)		1.00	(Ref.)		1.00	(Ref.)	
180–299 min/day	204/1788	34.2	0.95	(0.77–1.18)	0.660	0.93	(0.75–1.16)	0.531	0.93	(0.75–1.15)	0.518
300–479 min/day	269/2070	39.5	1.03	(0.84–1.26)	0.789	0.97	(0.79–1.19)	0.786	0.96	(0.78–1.17)	0.669
≥480 min/day	382/2316	50.9	1.25	(1.03–1.51)	0.024	1.13	(0.93–1.37)	0.219	1.10	(0.90–1.33)	0.351
	1001/7480	40.7	Trend		0.003	Trend		0.073	Trend		0.149
Non-frail (*n* = 5,743)											
MVPA											
Low (<600 METs·min/wk)	146/1108	40.0	1.00	(Ref.)		1.00	(Ref.)		1.00	(Ref.)	
Moderate (600-3000METs·min/wk)	250/2859	25.6	0.67	(0.54–0.82)	<0.001	0.70	(0.57–0.87)	0.001	0.71	(0.57–0.87)	0.001
High (>3,000 METs·min/wk)	158/1776	26.3	0.72	(0.57–0.90)	0.004	0.76	(0.61–0.96)	0.020	0.77	(0.61–0.97)	0.026
	554/5743	28.5	Trend		0.008	Trend		0.032	Trend		0.037
ST											
<180 min/day	87/1044	24.7	1.00	(Ref.)		1.00	(Ref.)		1.00	(Ref.)	
180–299 min/day	129/1435	26.4	0.99	(0.75–1.30)	0.922	0.94	(0.72–1.24)	0.673	0.93	(0.71–1.23)	0.610
300–479 min/day	146/1597	27.0	0.95	(0.73–1.24)	0.720	0.91	(0.70–1.20)	0.512	0.91	(0.69–1.19)	0.469
≥480 min/day	192/1667	34.3	1.14	(0.89–1.47)	0.304	1.10	(0.85–1.43)	0.459	1.07	(0.83–1.39)	0.591
	554/5743	28.5	Trend		0.241	Trend		0.329	Trend		0.402
Frail (*n* = 1737)											
MVPA											
Low (<600 METs·min/wk)	239/813	101.8	1.00	(Ref.)		1.00	(Ref.)		1.00	(Ref.)	
Moderate (600-3000METs·min/wk)	162/653	81.5	0.81	(0.67–0.99)	0.043	0.73	(0.60–0.90)	0.004	0.74	(0.60–0.91)	0.005
High (>3,000 METs·min/wk)	46/271	53.5	0.55	(0.40–0.76)	<0.001	0.50	(0.36–0.69)	<0.001	0.51	(0.37–0.71)	<0.001
	447/1737	86.0	Trend		<0.001	Trend		<0.001	Trend		<0.001
ST											
<180 min/day	59/262	73.6	1.00	(Ref.)		1.00	(Ref.)		1.00	(Ref.)	
180–299 min/day	75/353	69.6	0.91	(0.65–1.28)	0.596	0.84	(0.59–1.19)	0.317	0.84	(0.59–1.20)	0.345
300–479 min/day	123/473	87.4	1.05	(0.77–1.44)	0.753	1.02	(0.74–1.41)	0.905	0.98	(0.71–1.35)	0.906
≥480 min/day	190/649	99.5	1.18	(0.88–1.58)	0.269	1.16	(0.86–1.57)	0.348	1.10	(0.81–1.50)	0.526
	447/1737	86.0	Trend		0.088	Trend		0.075	Trend		0.174

Model 1: Adjusted for baseline age and sex.

Model 2: Adjusted for variables in Model 1 plus district, living situation, marital status, education, equivalent income, body mass index, hypertension, dyslipidemia, heart disease, stroke, diabetes mellitus, cancer, alcohol drinking status, smoking status, lower-back pain, knee pain, and frailty.

Model 3: For MVPA, adjusted for variables in Model 2 plus ST. For ST, adjusted for variables in Model 2 plus MVPA.

In stratified analyses for participants with/without frailty status, frailty was excluded from covariates.

CI = confidence interval; FD = functional disability; HR = hazard ratio; METs = metabolic equivalents; MVPA = moderate-to-vigorous physical activity; PY = person-years; ST = sedentary time.

**Figure 2:**
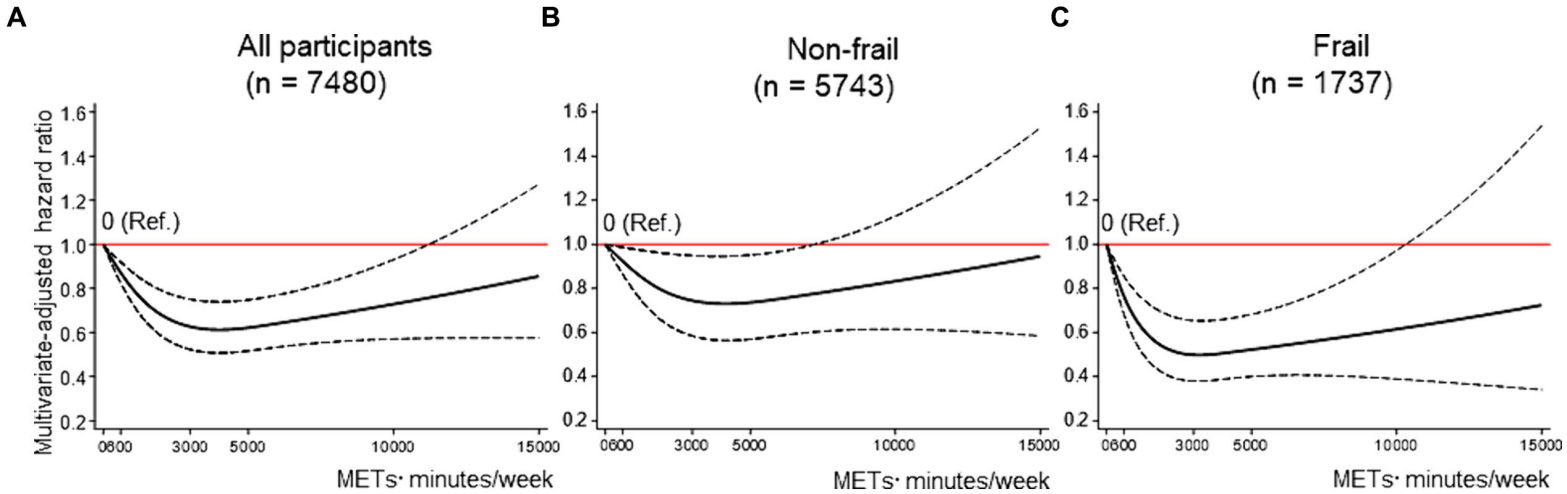
Dose–response association between MVPA and FD. **(A–C)** shows the association between MVPA and FD among all participants **(A)**, non-frail older adults **(B)**, and frail older adults **(C)**, modeled by restricted cubic splines. MVPA and ST were mutually adjusted, as were baseline age, sex, district, living situation, marital status, education, equivalent income, body mass index, hypertension, dyslipidemia, heart disease, stroke, diabetes mellitus, cancer, alcohol drinking status, smoking status, lower back pain, knee pain, and frailty [in the stratified analyses **(B,C)**, frailty was excluded from covariates]. The reference value for MVPA was 0 METs■min/week. Solid lines indicate hazard ratios for FD. Dashed lines indicate 95% confidence intervals. FD = functional disability; METs = metabolic equivalent; MVPA = moderate-to-vigorous physical activity; ST = sedentary time.

Figure 2 displays the dose–response curves of MVPA with FD in the fully adjusted model. Three knots were adopted for all restricted spline curves. Among all participants, compared to no MVPA (the reference), the HRs for FD decreased linearly up to approximately 2000 METs■min/week of MVPA, and the lowest HRs (HR: 0.61, 95% CI: 0.51–0.74) was reached at around 3,000–4,000 METs■min/week (Figure 2A). These lower HRs became modest above that MVPA, and the results indicated increased uncertainty, as evidenced by the wide 95% CIs at MVPA levels of approximately ≥11,000 METs■min/week. Among non-frail older adults, although the shape of the dose–response curve was similar to the results for all participants, the strength of the association tended to be attenuated (e.g., the lowest HR: 0.73, 95% CI: 0.56–0.95 at around 3,500–4,500 METs■min/week) (Figure 2B). Among frail older adults, the HRs for FD reduced more steeply and linearly up to approximately 1,500 METs■min/week of MVPA, and the lowest HRs (HR: 0.50, 95% CI: 0.38–0.65) were reached at around 2,500–3,000 METs■min/week (Figure 2C). The results of the sensitivity analyses showed that although the strength of any association weakened, the shapes of the dose–response curves remained unchanged compared to those of the primary analyses (Supplementary Figure 1).

**Figure 3:**
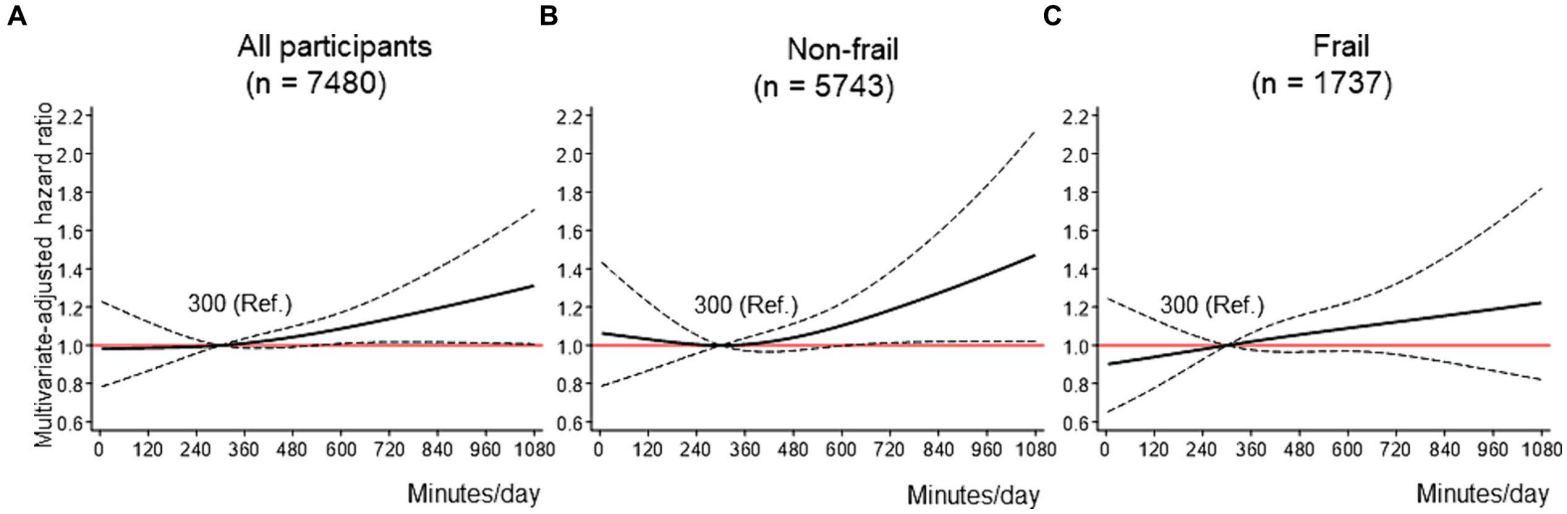
Dose–response association between ST and FD. **(A-C)** shows the association between ST and FD among all participants **(A)**, non-frail older adults **(B)**, and frail older adults **(C)**, modeled by restricted cubic splines. ST and MVPA were mutually adjusted, as well as baseline age, sex, district, living situation, marital status, education, equivalent income, body mass index, hypertension, dyslipidemia, heart disease, stroke, diabetes mellitus, cancer, alcohol drinking status, smoking status, lower back pain, knee pain, and frailty (in the stratified analyses **(B,C)**, frailty was excluded from covariates). The reference value for the ST was 300 min/day. Solid lines indicate hazard ratios for FD. Dashed lines indicate 95% confidence intervals. FD = functional disability; METs = metabolic equivalent; MVPA = moderate-to-vigorous physical activity; ST = sedentary time.

Figure 3 displays the dose–response curves of ST with FD in the fully adjusted model. Three knots were adopted for all restricted spline curves. Among all participants, compared with 300 min/day of ST, the HRs for FD increased linearly as ST reached approximately 600 min/day or more, with a maximum HR of 1.31 (95% CI: 1.01–1.71) for 1,080 min/day (Figure 3A). This trend was particularly pronounced among non-frail older adults, with a maximum HR of 1.47 (95% CI: 1.02–2.12) for 1,080 min/day (Figure 3B). Among frail older adults, although a trend toward a linear association between ST and FD was observed, the association was not statistically significant (Figure 3C). The sensitivity analysis results showed that the shapes of the dose–response curves were not substantially different from those of the primary analyses (Supplementary Figure 2).

## 4 Discussion

Regarding MVPA, a consistent, nonlinear, and significant inverse dose–response association was identified for FD risk, regardless of frailty status. The association between MVPA and FD tended to be stronger in frail older adults than in non-frail older adults, and a slight increase in MVPA (up to approximately 1,500 METs■min/week) substantially decreased HR for FD even in frail older adults. Regarding ST, a significant nonlinear positive dose–response association was confirmed with FD risk among non-frail older adults. The HR for FD increased significantly at approximately 600 min/day or more compared to that at 300 min/day. However, no significant dose–response association between ST and FD was observed among frail older adults.

Previous large-scale studies examining dose–response relationships between PA and health outcomes suggested that even a small increase in PA among physically inactive individuals reduced the risk of cardiovascular disease, cancer, and mortality (5–8, 22, 34, 35). For instance, a dose–response meta-analysis of eight studies (36,383 participants) using accelerometry-measured PA showed that individuals performing 20–30 min/day of MVPA had an approximately 60% risk reduction for all-cause mortality (35). Another dose–response meta-analysis of 94 cohorts (30 million participants) revealed that 75 min/week of MPA (approximately 11 min/day) significantly reduced the risk of mortality, cardiovascular disease, and cancer (7). Similar to the results of these studies (5–8, 22, 34, 35), the only dose–response analysis with FD as an outcome showed that a higher MVPA, regardless of about of <10 or ≥ 10 min, significantly reduced the risk of FD (10). Our findings prove that these relationships are consistent, even in frail older adults.

However, our finding is somewhat contrary to the results of a recent study (13). A 3.38-year cohort study that examined dose–response relationships between objectively measured daily steps and mortality (13) reported that frail older adults may require more daily steps to achieve an inverse association with mortality than non-frail older adults. The daily steps of older adults include not only MVPA but also LPA of <3 METs (36). As slow walking speed is an important phenotype of frailty (26), the daily steps of frail older adults may contain more LPA than those of non-frail older adults (e.g., slow walking [approximately 0.89 m/s] is equivalent to 2.8 METs (37)). Several Japanese cohort studies in older adults have reported that total LPA is not significantly associated with a reduced risk of FD (10, 38). Therefore, the study in question (13) may have concluded that frail older adults require more daily steps than non-frail ones to reduce the risk of mortality. It is essential to increase MVPA, even slightly, to reduce the risk of FD in frail older adults.

Our results emphasize the significance of avoiding prolonged ST independent of MVPA among non-frail older adults, although this alone is insufficient to reduce the risk of FD among frail older adults. Previous studies (11, 39, 40) have reported mixed results regarding whether the relationship between ST and disability is independent of PA. One study (39) showed that the association between ST and mobility disability remained statistically significant, even after adjusting for PA, whereas two studies (11, 40) showed that the association between ST and functional limitations/FD was not statistically independent of MVPA, suggesting that decreased ST may be replaced by increased MVPA. This discrepancy in previous results may be partially explained by the presence or absence of frailty. The significant positive dose–response association between ST and FD among frail older adults completely disappeared after adjusting for MVPA, indicating that the association was not independent of MVPA in frail older adults. Previous studies reported that replacing 10 min of ST with MVPA led to a 12–13% reduction in FD risk, whereas replacing ST with LPA did not consistently reduce the risk of FD (11, 41). Based on these and our results, reducing ST alone may be insufficient to reduce the risk of FD; it is important to replace ST with MVPA, but not LPA, especially in frail older adults.

FD risk did not significantly increase even at high levels of MVPA, regardless of frailty status. Although our dose–response curve between MVPA and FD risk showed an inverse J-shaped association, this may be attributed to reverse causation bias due to the short follow-up period (42, 43) and regression dilution bias due to measurement error based on a single assessment at baseline (43). Individuals who have pre-existing conditions that elevate their risk may become less active, which could result in an overestimation of the true inverse association, particularly between lower levels of MVPA and risk (42). Furthermore, the association between a single measure of PA and health outcomes is more likely to be inverse J-shaped or somewhat U-shaped compared to repeated measures of PA (43). Therefore, the inverse J-shaped association observed in this study may be due to bias rather than actual biological effects. However, there are many unknown aspects regarding the impact of high-volume PA, particularly in older adults (9). Since the IPAQ-SF tends to overestimate PA compared to more accurate objective devices (19), engaging in slightly lower MVPA than the MVPA values obtained in this study may be safe and reasonable for older adults.

This study had some limitations. First, the main limitation was the cognitive bias caused by assessing MVPA and ST using the IPAQ-SF. This bias overestimates MVPA and underestimates ST (19, 44). The MVPA and ST values obtained in our study should be interpreted considering these biases. Moreover, it should be noted that the IPAQ-short questionnaire is unable to assess the levels of LPA. Thus, comparing the effects of LPA and ST on FD remains a challenge to be addressed in future studies. Seconds, it is important to address the issue of selection bias. In this study, there was a notable concern regarding the incidence rate of FD among the 3,987 individuals who had missing data on IPAQ-SF and/or frailty. The incidence rate was found to be significantly higher in this subgroup than in the participants who were included in the analysis (as shown in Supplementary Table 3). Third, our study focused solely on older adults residing in a metropolitan area, which could potentially restrict generalization. Fourth, the follow-up period in this study was relatively short. However, follow-up after the endpoint set in our study was concerning because the novel coronavirus disease pandemic significantly reduced PA (45) and new LTCI applications (30). Therefore, this was set as the minimum required follow-up period. Although we conducted sensitivity analyses that excluded the occurrence of FDs during the first year, the possibility of reverse causation cannot be entirely ruled out. Fifth, an older adult, his/her family member, or caregiver must contact the municipal government to officially certify their FDs (16). Therefore, some individuals with FDs may not have reported themselves, leading to an underestimation of the incidence of FD (detection bias). Finally, this study utilized data from a community-wide intervention that included PA promotion (15). Although the intervention did not have an impact on population-level frailty at 2 years (15) or incident FD during the present follow-up period (data not shown), walking time in the intervention subgroup improved at the population-level (15). MVPA was assessed only at baseline, and we were unable to evaluate any changes in MVPA during the follow-up period. If MVPA had increased more during the subsequent follow-up period after 2 years, the association between MVPA and FD risk shown in our study might have been underestimated.

Although our study has limitations, it is strengthened by the inclusion of a large sample size of randomly recruited participants and high follow-up rates. The findings of our study can aid in developing future PA guidelines for older adults.

In conclusion, a significant inverse nonlinear dose–response association between MVPA and FD risk was identified regardless of frailty status. Even a slight increase in MVPA (up to approximately 2000 METs■min/week) led to a reduction in the FD risk, and this was more pronounced among frail older adults. Although a significant positive nonlinear dose–response association of ST with FD risk, independent of MVPA, was identified among non-frail older adults, this relationship was not significant among frail older adults. In non-frail older adults, reducing prolonged ST and increasing MVPA may be important for preventing FD. For frail older adults, to prevent FD, it may be important to not only reduce ST but to replace it with MVPA, even if by only small increments.

## Data availability statement

The raw data supporting the conclusions of this article will be made available by the authors, without undue reservation.

## Ethics statement

The Ethical Committee of the Tokyo Metropolitan Institute for Geriatrics and Gerontology approved this study on June 1, 2016 (reference number: 8). All participants were informed that their participation in this study was voluntary, and they provided informed consent.

## Author contributions

SSe: Writing – review & editing, Writing – original draft, Supervision, Project administration, Investigation, Funding acquisition, Formal analysis, Conceptualization. TA: Writing – review & editing. YN: Writing – review & editing. TH: Writing – review & editing, Investigation. SSh: Writing – review & editing, Supervision, Project administration, Funding acquisition, Conceptualization. AK: Writing – review & editing, Supervision, Project administration. YF: Writing – review & editing, Supervision, Project administration.
