# Supplementary material for: Dose–response associations between physical activity and sedentary time with functional disability in older adults with or without frailty: a prospective cohort study
Source: Front Public Health. 2024 Apr 24;12:1357618. doi: 10.3389/fpubh.2024.1357618 (PMC11076770; doi:10.3389/fpubh.2024.1357618)
Supplement: Supplementary file 1 [file Table_1.DOCX]

Supplementary Material

# Supplementary Figures and Tables

## Supplementary Tables

| **Supplementary Table 1**. Baseline characteristics of the study population without frailty according to FD status | | | | | | | |
| --- | --- | --- | --- | --- | --- | --- | --- |
|  | All | | Without  incident FD | | Incident FD | | *P* |
|  | (n = 5743) | | (n = 5189) | | (n = 554) | |  |
| **VPA (min/wk),**  **median (interquartile range)** | 0 | (0-100) | 0 | (0-105) | 0 | (0-80) | 0.169 |
| **MPA (min/wk),**  **median (interquartile range)** | 0 | (0-150) | 0 | (0-150) | 0 | (0-120) | 0.005 |
| **Walking time (min/wk),**  **median (interquartile range)** | 350 | (150-630) | 360 | (150-630) | 300 | (120-600) | 0.016 |
| **MVPA (METs･min/wk),**  **median (interquartile range)** | 1782 | (798-3573) | 1788 | (855-3600) | 1485 | (594-3348) | 0.001 |
| Low (<600 METs・min/wk), n (%) | 1108 | (19.3) | 962 | (18.5) | 146 | (26.4) |  |
| Moderate (600-3000 METs・min/wk), n (%) | 2859 | (49.8) | 2609 | (50.3) | 250 | (45.1) | <0.001 |
| High (>3000 METs・min/wk), n (%) | 1776 | (30.9) | 1618 | (31.2) | 158 | (28.5) |  |
| **ST (min/day),**  **median (interquartile range)** | 300 | (180-480) | 300 | (180-480) | 300 | (180-540) | <0.001 |
| <180, n (%) | 1044 | (18.2) | 957 | (18.4) | 87 | (15.7) | 0.019 |
| 180-299, n (%) | 1435 | (25.0) | 1306 | (25.2) | 129 | (23.3) |  |
| 300-479, n (%) | 1597 | (27.8) | 1451 | (28.0) | 146 | (26.4) |  |
| ≥480, n (%) | 1667 | (29.0) | 1475 | (28.4) | 192 | (34.7) |  |
| **Age (years), mean (SD)** | 73.3 | (5.4) | 72.8 | (5.3) | 77.3 | (4.9) | <0.001 |
| **Sex (men), n (%)** | 2744 | (47.8) | 2487 | (47.9) | 257 | (46.4) | 0.491 |
| **Living alone, n (%)** | 1091 | (19.0) | 976 | (18.8) | 115 | (20.8) | 0.539 |
| **Marital status, n (%)** |  |  |  |  |  |  | 0.002 |
| Married | 3986 | (69.4) | 3624 | (69.8) | 362 | (65.3) |  |
| Widowed or divorced | 1281 | (22.3) | 1127 | (21.7) | 154 | (27.8) |  |
| Never married | 422 | (7.4) | 392 | (7.6) | 30 | (5.4) |  |
| **Education, n (%)** |  |  |  |  |  |  | <0.001 |
| Junior high school graduation | 1057 | (18.4) | 922 | (17.8) | 135 | (24.4) |  |
| High school graduation | 2183 | (38.0) | 1976 | (38.1) | 207 | (37.4) |  |
| Junior college/vocational college/ college/graduate school graduation | 2383 | (41.5) | 2188 | (42.2) | 195 | (35.2) |  |
| Other/missing | 120 | (2.1) | 103 | (2.0) | 17 | (3.1) |  |
| **Equivalent income, n (%)** |  |  |  |  |  |  | 0.004 |
| <2.0 million yen | 884 | (15.4) | 783 | (15.1) | 101 | (18.2) |  |
| 2.0-3.99 million yen | 1860 | (32.4) | 1657 | (31.9) | 203 | (36.6) |  |
| ≥4.0 million yen | 1828 | (31.8) | 1669 | (32.2) | 159 | (28.7) |  |
| Unknown/missing | 1171 | (20.4) | 1080 | (20.8) | 91 | (16.4) |  |
| **BMI (kg/m^2^), mean (SD)** | 22.7 | (3.0) | 22.7 | (2.9) | 22.7 | (3.5) | 0.497 |
| <18.5, n (%) | 435 | (7.6) | 375 | (7.2) | 60 | (10.8) |  |
| 18.5-24.9, n (%) | 4142 | (72.1) | 3775 | (72.8) | 367 | (66.3) | 0.001 |
| ≥25, n (%) | 1146 | (20.0) | 1023 | (19.7) | 123 | (22.2) |  |
| **Hypertension, n (%)** | 2885 | (50.2) | 2577 | (49.7) | 308 | (55.6) | 0.005 |
| **Dyslipidemia, n (%)** | 2380 | (41.4) | 2171 | (41.8) | 209 | (37.7) | 0.010 |
| **Heart disease, n (%)** | 1113 | (19.4) | 978 | (18.9) | 135 | (24.4) | 0.003 |
| **Stroke, n (%)** | 298 | (5.2) | 257 | (5.0) | 41 | (7.4) | 0.013 |
| **Diabetes mellitus, n (%)** | 903 | (15.7) | 795 | (15.3) | 108 | (19.5) | 0.002 |
| **Cancer, n (%)** | 826 | (14.4) | 715 | (13.8) | 111 | (20.0) | <0.001 |
| **Alcohol drinking status (current), n (%)** | 3379 | (58.8) | 3104 | (59.8) | 275 | (49.6) | <0.001 |
| **Smoking status (current), n (%)** | 658 | (11.5) | 609 | (11.7) | 49 | (8.8) | 0.055 |
| **Lower back pain, n (%)** | 1924 | (33.5) | 1692 | (32.6) | 232 | (41.9) | <0.001 |
| **Knee pain, n (%)** | 1565 | (27.3) | 1360 | (26.2) | 205 | (37.0) | <0.001 |
| BMI = body mass index; FD = functional disability; METs = metabolic equivalents; MPA = moderate physical activity; MVPA = moderate-to-vigorous physical activity; SD = standard deviation; ST = sedentary time; VPA = vigorous physical activity. | | | | | | | |

| **Supplementary Table 2**. Baseline characteristics of the study population with frailty according to FD status | | | | | | | |
| --- | --- | --- | --- | --- | --- | --- | --- |
|  | All | | Without  incident FD | | Incident FD | | *P* |
|  | (n = 1737) | | (n = 1290) | | (n = 447) | |  |
| **VPA (min/wk),**  **median (interquartile range)** | 0 | (0-0) | 0 | (0-0) | 0 | (0-0) | 0.078 |
| **MPA (min/wk),**  **median (interquartile range)** | 0 | (0-0) | 0 | (0-0) | 0 | (0-0) | 0.022 |
| **Walking time (min/wk),**  **median (interquartile range)** | 160 | (20-375) | 180 | (40-420) | 120 | (0-300) | <0.001 |
| **MVPA (METs･min/wk),**  **median (interquartile range)** | 693 | (132-2079) | 792 | (198-2106) | 528 | (0-1390) | <0.001 |
| Low (<600 METs・min/wk), n (%) | 813 | (46.8) | 574 | (44.5) | 239 | (53.5) |  |
| Moderate (600-3000METs・min/wk), n (%) | 653 | (37.6) | 491 | (38.1) | 162 | (36.2) | <0.001 |
| High (>3000 METs・min/wk), n (%) | 271 | (15.6) | 225 | (17.4) | 46 | (10.3) |  |
| **ST (min/day),**  **median (interquartile range)** | 360 | (210-540) | 330 | (180-540) | 360 | (240-600) | 0.002 |
| <180, n (%) | 262 | (15.1) | 203 | (15.7) | 59 | (13.2) | 0.024 |
| 180-299, n (%) | 353 | (20.3) | 278 | (21.6) | 75 | (16.8) |  |
| 300-479, n (%) | 473 | (27.2) | 350 | (27.1) | 123 | (27.5) |  |
| ≥480, n (%) | 649 | (37.4) | 459 | (35.6) | 190 | (42.5) |  |
| **Age (years), mean (SD)** | 74.9 | (5.6) | 74.1 | (5.5) | 77.3 | (5.0) | <0.001 |
| **Sex (men), n (%)** | 1051 | (60.5) | 777 | (60.2) | 274 | (61.3) | 0.691 |
| **Living alone, n (%)** | 400 | (23.0) | 285 | (22.1) | 115 | (25.7) | 0.246 |
| **Marital status, n (%)** |  |  |  |  |  |  | 0.033 |
| Married | 1105 | (63.6) | 827 | (64.1) | 278 | (62.2) |  |
| Widowed or divorced | 452 | (26.0) | 320 | (24.8) | 132 | (29.5) |  |
| Never married | 154 | (8.9) | 126 | (9.8) | 28 | (6.3) |  |
| **Education, n (%)** |  |  |  |  |  |  | 0.812 |
| Junior high school graduation | 505 | (29.1) | 368 | (28.5) | 137 | (30.7) |  |
| High school graduation | 641 | (36.9) | 481 | (37.3) | 160 | (35.8) |  |
| Junior college/vocational college/ college/graduate school graduation | 526 | (30.3) | 394 | (30.5) | 132 | (29.5) |  |
| Other/missing | 65 | (3.7) | 47 | (3.6) | 18 | (4.0) |  |
| **Equivalent income, n (%)** |  |  |  |  |  |  | 0.016 |
| <2.0 million yen | 318 | (18.3) | 216 | (16.7) | 102 | (22.8) |  |
| 2.0-3.99 million yen | 773 | (44.5) | 575 | (44.6) | 198 | (44.3) |  |
| ≥4.0 million yen | 449 | (25.9) | 351 | (27.2) | 98 | (21.9) |  |
| Unknown/missing | 197 | (11.3) | 148 | (11.5) | 49 | (11.0) |  |
| **BMI (kg/m^2^), mean (SD)** | 22.9 | (3.5) | 23.1 | (3.4) | 22.4 | (3.6) | <0.001 |
| <18.5, n (%) | 151 | (8.7) | 93 | (7.2) | 58 | (13.0) |  |
| 18.5-24.9, n (%) | 1136 | (65.4) | 851 | (66.0) | 285 | (63.8) | 0.001 |
| ≥25, n (%) | 432 | (24.9) | 334 | (25.9) | 98 | (21.9) |  |
| **Hypertension, n (%)** | 1056 | (60.8) | 782 | (60.6) | 274 | (61.3) | 0.705 |
| **Dyslipidemia, n (%)** | 745 | (42.9) | 574 | (44.5) | 171 | (38.3) | 0.009 |
| **Heart disease, n (%)** | 539 | (31.0) | 376 | (29.2) | 163 | (36.5) | 0.003 |
| **Stroke, n (%)** | 218 | (12.6) | 145 | (11.2) | 73 | (16.3) | 0.016 |
| **Diabetes mellitus, n (%)** | 412 | (23.7) | 299 | (23.2) | 113 | (25.3) | 0.615 |
| **Cancer, n (%)** | 372 | (21.4) | 259 | (20.1) | 113 | (25.3) | 0.024 |
| **Alcohol drinking status (current), n (%)** | 909 | (52.3) | 694 | (53.8) | 215 | (48.1) | <0.001 |
| **Smoking status (current), n (%)** | 300 | (17.3) | 222 | (17.2) | 78 | (17.5) | 0.979 |
| **Lower back pain, n (%)** | 875 | (50.4) | 642 | (49.8) | 233 | (52.1) | 0.611 |
| **Knee pain, n (%)** | 679 | (39.1) | 499 | (38.7) | 180 | (40.3) | 0.304 |
| BMI = body mass index; FD = functional disability; METs = metabolic equivalents; MPA = moderate physical activity; MVPA = moderate-to-vigorous physical activity; SD = standard deviation; ST = sedentary time; VPA = vigorous physical activity. | | | | | | | |

| **Supplementary Table 3**. Baseline characteristics of the study population, encompassing those included and excluded from the analysis | | | | | | | |
| --- | --- | --- | --- | --- | --- | --- | --- |
|  | All | | Included in the analysis | | Excluded in the analysis | | *P* |
|  | (n = 11467) | | (n = 7480) | | (n = 3987) | |  |
| **Incidence of FD** | 1801 | (15.7) | 1001 | (13.4) | 800 | (20.1) | <0.001 |
| **Age (years), mean (SD)** | 74.2 | 5.5 | 73.7 | (5.5) | 75.2 | (5.3) | <0.001 |
| **Sex (men), n (%)** | 5579 | (48.7) | 3795 | (50.7) | 1784 | (44.8) | <0.001 |
| **Living alone, n (%)** | 2311 | (20.2) | 1491 | (19.9) | 820 | (20.6) | <0.001 |
| **Marital status, n (%)** |  |  |  |  |  |  | <0.001 |
| Married | 7496 | (65.4) | 5091 | (68.1) | 2405 | (60.3) |  |
| Widowed or divorced | 2874 | (25.1) | 1733 | (23.2) | 1141 | (28.6) |  |
| Never married | 812 | (7.1) | 576 | (7.7) | 236 | (5.9) |  |
| **Education, n (%)** |  |  |  |  |  |  | <0.001 |
| Junior high school graduation | 2848 | (24.8) | 1562 | (20.9) | 1286 | (32.3) |  |
| High school graduation | 4327 | (37.7) | 2824 | (37.8) | 1503 | (37.7) |  |
| Junior college/vocational college/ college/graduate school graduation | 3884 | (33.9) | 2909 | (38.9) | 975 | (24.5) |  |
| Other/missing | 408 | (3.6) | 185 | (2.5) | 223 | (5.6) |  |
| **Equivalent income, n (%)** |  |  |  |  |  |  | <0.001 |
| <2.0 million yen | 2315 | (20.2) | 1202 | (16.1) | 1113 | (27.9) |  |
| 2.0-3.99 million yen | 4181 | (36.5) | 2633 | (35.2) | 1548 | (38.8) |  |
| ≥4.0 million yen | 3140 | (27.4) | 2277 | (30.4) | 863 | (21.7) |  |
| Unknown/missing | 1831 | (16.0) | 1368 | (18.3) | 463 | (11.6) |  |
| **BMI (kg/m^2^), mean (SD)** | 22.7 | (3.2) | 22.7 | (3.1) | 22.7 | (3.3) | 0.674 |
| <18.5, n (%) | 904 | (7.9) | 586 | (7.8) | 318 | (8.0) |  |
| 18.5-24.9, n (%) | 7965 | (69.5) | 5278 | (70.6) | 2687 | (67.4) | <0.001 |
| ≥25, n (%) | 2429 | (21.2) | 1578 | (21.1) | 851 | (21.3) |  |
| **Hypertension, n (%)** | 5936 | (51.8) | 3941 | (52.7) | 1995 | (50.0) | <0.001 |
| **Dyslipidemia, n (%)** | 4514 | (39.4) | 3125 | (41.8) | 1389 | (34.8) | <0.001 |
| **Heart disease, n (%)** | 2358 | (20.6) | 1652 | (22.1) | 706 | (17.7) | <0.001 |
| **Stroke, n (%)** | 806 | (7.0) | 516 | (6.9) | 290 | (7.3) | <0.001 |
| **Diabetes mellitus, n (%)** | 2024 | (17.7) | 1315 | (17.6) | 709 | (17.8) | <0.001 |
| **Cancer, n (%)** | 1776 | (15.5) | 1198 | (16.0) | 578 | (14.5) | <0.001 |
| **Alcohol drinking status (current), n (%)** | 6230 | (54.3) | 4288 | (57.3) | 1942 | (48.7) | <0.001 |
| **Smoking status (current), n (%)** | 1433 | (12.5) | 958 | (12.8) | 475 | (11.9) | <0.001 |
| **Lower back pain, n (%)** | 4212 | (36.7) | 2799 | (37.4) | 1413 | (35.4) | <0.001 |
| **Knee pain, n (%)** | 3461 | (30.2) | 2244 | (30.0) | 1217 | (30.5) | <0.001 |
| BMI = body mass index; FD = functional disability; SD = standard deviation. | | | | | | | |

| **Supplementary Table 4**. Multivariate-adjusted HRs and 95% CIs of MVPA and ST for incident FD with multiple imputation of covariates | | | | | | | | | | | | | |
| --- | --- | --- | --- | --- | --- | --- | --- | --- | --- | --- | --- | --- | --- |
| Variables | Number of events per participants | Incidence rate per 1000 PY | Model 1 | | |  | Model 2 | | |  | Model 3 | | |
|  |  |  | HR | (95% CI) | *P* |  | HR | (95% CI) | *P* |  | HR | (95% CI) | *P* |
| **All participants (n = 7480)** |  |  |  |  |  |  |  |  |  |  |  |  |  |
| **MVPA** |  |  |  |  |  |  |  |  |  |  |  |  |  |
| Low (<600 METs･min/wk) | 385/1921 | 64.2 | 1.00 | (Ref.) |  |  | 1.00 | (Ref.) |  |  | 1.00 | (Ref.) |  |
| Moderate (600-3000 METs･min/wk) | 412/3512 | 35.1 | 0.59 | (0.51-0.68) | <0.001 |  | 0.74 | (0.64-0.85) | <0.001 |  | 0.74 | (0.64-0.86) | <0.001 |
| High (>3000 METs･min/wk) | 204/2047 | 29.7 | 0.52 | (0.44-0.62) | <0.001 |  | 0.68 | (0.57-0.81) | <0.001 |  | 0.68 | (0.57-0.82) | <0.001 |
|  | 1001/7480 | 40.7 | Trend | | <0.001 |  | Trend | | <0.001 |  | <0.001 | | <0.001 |
| **ST** |  |  |  |  |  |  |  |  |  |  |  |  |  |
| <180 min/day | 146/1306 | 33.8 | 1.00 | (Ref.) |  |  | 1.00 | (Ref.) |  |  | 1.00 | (Ref.) |  |
| 180-299 min/day | 204/1788 | 34.2 | 0.95 | (0.77-1.18) | 0.660 |  | 0.94 | (0.75-1.16) | 0.538 |  | 0.93 | (0.75-1.16) | 0.524 |
| 300-479 min/day | 269/2070 | 39.5 | 1.03 | (0.84-1.26) | 0.789 |  | 0.97 | (0.79-1.19) | 0.798 |  | 0.96 | (0.78-1.17) | 0.686 |
| ≥480 min/day | 382/2316 | 50.9 | 1.25 | (1.03-1.51) | 0.024 |  | 1.12 | (0.93-1.37) | 0.236 |  | 1.09 | (0.90-1.33) | 0.372 |
|  | 1001/7480 | 40.7 | Trend | | 0.003 |  | Trend | | 0.083 |  | Trend | | 0.166 |
| **Non-frail (n = 5743)** |  |  |  |  |  |  |  |  |  |  |  |  |  |
| **MVPA** |  |  |  |  |  |  |  |  |  |  |  |  |  |
| Low (<600 METs･min/wk) | 146/1108 | 40.0 | 1.00 | (Ref.) |  |  | 1.00 | (Ref.) |  |  | 1.00 | (Ref.) |  |
| Moderate (600-3000 METs･min/wk) | 250/2859 | 25.6 | 0.67 | (0.54-0.82) | <0.001 |  | 0.71 | (0.57-0.87) | 0.001 |  | 0.71 | (0.58-0.88) | 0.001 |
| High (>3000 METs･min/wk) | 158/1776 | 26.3 | 0.72 | (0.57-0.90) | 0.004 |  | 0.76 | (0.60-0.96) | 0.019 |  | 0.77 | (0.61-0.97) | 0.024 |
|  | 554/5743 | 28.5 | Trend | | 0.008 |  | Trend | | 0.031 |  | Trend | | 0.036 |
| **ST** |  |  |  |  |  |  |  |  |  |  |  |  |  |
| <180 min/day | 87/1044 | 24.7 | 1.00 | (Ref.) |  |  | 1.00 | (Ref.) |  |  | 1.00 | (Ref.) |  |
| 180-299 min/day | 129/1435 | 26.4 | 0.99 | (0.75-1.30) | 0.922 |  | 0.95 | (0.72-1.24) | 0.686 |  | 0.93 | (0.71-1.23) | 0.620 |
| 300-479 min/day | 146/1597 | 27.0 | 0.95 | (0.73-1.24) | 0.720 |  | 0.91 | (0.70-1.19) | 0.496 |  | 0.91 | (0.69-1.18) | 0.455 |
| ≥480 min/day | 192/1667 | 34.3 | 1.14 | (0.89-1.47) | 0.304 |  | 1.10 | (0.85-1.42) | 0.480 |  | 1.07 | (0.83-1.39) | 0.605 |
|  | 554/5743 | 28.5 | Trend | | 0.241 |  | Trend | | 0.356 |  | Trend | | 0.431 |
| **Frail (n = 1737)** |  |  |  |  |  |  |  |  |  |  |  |  |  |
| **MVPA** |  |  |  |  |  |  |  |  |  |  |  |  |  |
| Low (<600 METs･min/wk) | 239/813 | 101.8 | 1.00 | (Ref.) |  |  | 1.00 | (Ref.) |  |  | 1.00 | (Ref.) |  |
| Moderate (600-3000 METs･min/wk) | 162/653 | 81.5 | 0.81 | (0.67-0.99) | 0.043 |  | 0.75 | (0.61-0.92) | 0.006 |  | 0.76 | (0.62-0.93) | 0.008 |
| High (>3000 METs･min/wk) | 46/271 | 53.5 | 0.55 | (0.40-0.76) | <0.001 |  | 0.50 | (0.36-0.69) | <0.001 |  | 0.51 | (0.37-0.71) | <0.001 |
|  | 447/1737 | 86.0 | Trend | | <0.001 |  | Trend | | <0.001 |  | Trend | | <0.001 |
| **ST** |  |  |  |  |  |  |  |  |  |  |  |  |  |
| <180 min/day | 59/262 | 73.6 | 1.00 | (Ref.) |  |  | 1.00 | (Ref.) |  |  | 1.00 | (Ref.) |  |
| 180-299 min/day | 75/353 | 69.6 | 0.91 | (0.65-1.28) | 0.596 |  | 0.85 | (0.60-1.21) | 0.370 |  | 0.86 | (0.60-1.22) | 0.397 |
| 300-479 min/day | 123/473 | 87.4 | 1.05 | (0.77-1.44) | 0.753 |  | 1.05 | (0.76-1.44) | 0.773 |  | 1.01 | (0.73-1.39) | 0.945 |
| ≥480 min/day | 190/649 | 99.5 | 1.18 | (0.88-1.58) | 0.269 |  | 1.15 | (0.85-1.56) | 0.364 |  | 1.10 | (0.81-1.49) | 0.546 |
|  | 447/1737 | 86.0 | Trend | | 0.088 |  | Trend | | 0.094 |  | Trend | | 0.208 |
| Model 1: Adjusted for baseline age and sex. | | | | | | | | | | | | | |
| Model 2: Adjusted for variables in Model 1 plus district, living situation, marital status, education, equivalent income, body mass index, hypertension, dyslipidemia, heart disease, stroke, diabetes mellitus, cancer, alcohol drinking status, smoking status, lower-back pain, knee pain, and frailty. | | | | | | | | | | | | | |
| Model 3: For MVPA, adjusted for variables in Model 2 plus ST. For ST, adjusted for variables in Model 2 plus MVPA. | | | | | | | | | | | | | |
| In stratified analyses for participants with/without frailty status, frailty was excluded from covariates. | | | | | | | | | | | | | |
| CI = confidence interval; FD = functional disability; HR = hazard ratio; METs = metabolic equivalents; MVPA = moderate-to-vigorous physical activity; PY = person-years; ST = sedentary time. | | | | | | | | | | | | | |

| **Supplementary Table 5**. Multivariate-adjusted HRs and 95% CIs of MVPA and ST for incident FD, excluding FD that occurred during the first year of follow-up. | | | | | | | | | | | | | |
| --- | --- | --- | --- | --- | --- | --- | --- | --- | --- | --- | --- | --- | --- |
| Variables | Number of events per participants | Incidence rate per 1000 PY | Model 1 | | |  | Model 2 | | |  | Model 3 | | |
|  |  |  | HR | (95% CI) | *P* |  | HR | (95% CI) | *P* |  | HR | (95% CI) | *P* |
| **All participants (n = 7199)** | | | | | | | | | | | | | |
| **MVPA** |  |  |  |  |  |  |  |  |  |  |  |  |  |
| Low (<600 METs･min/wk) | 264/1800 | 44.5 | 1.00 | (Ref.) |  |  | 1.00 | (Ref.) |  |  | 1.00 | (Ref.) |  |
| Moderate (600-3000 METs･min/wk) | 303/3403 | 26.0 | 0.61 | (0.52-0.73) | <0.001 |  | 0.74 | (0.62-0.78) | 0.001 |  | 0.75 | (0.63-0.89) | 0.001 |
| High (>3000 METs･min/wk) | 153/1996 | 22.4 | 0.55 | (0.45-0.67) | <0.001 |  | 0.68 | (0.55-0.84) | <0.001 |  | 0.69 | (0.56-0.85) | <0.001 |
|  | 720/7199 | 29.5 | Trend | | <0.001 |  | Trend | | <0.001 |  | Trend | | <0.001 |
| **ST** |  |  |  |  |  |  |  |  |  |  |  |  |  |
| <180 min/day | 108/1268 | 25.1 | 1.00 | (Ref.) |  |  | 1.00 | (Ref.) |  |  | 1.00 | (Ref.) |  |
| 180-299 min/day | 148/1732 | 25.0 | 0.93 | (0.73-1.19) | 0.570 |  | 0.91 | (0.71-1.16) | 0.443 |  | 0.90 | (0.70-1.16) | 0.430 |
| 300-479 min/day | 188/1989 | 27.8 | 0.97 | (0.77-1.23) | 0.811 |  | 0.92 | (0.72-1.17) | 0.498 |  | 0.91 | (0.71-1.15) | 0.426 |
| ≥480 min/day | 276/2210 | 37.0 | 1.23 | (0.99-1.54) | 0.068 |  | 1.11 | (0.88-1.39) | 0.378 |  | 1.08 | (0.86-1.35) | 0.527 |
|  | 720/7199 | 29.5 | Trend | | 0.014 |  | Trend | | 0.162 |  | Trend | | 0.265 |
| **Non-frail (n = 5616)** |  |  |  |  |  |  |  |  |  |  |  |  |  |
| **MVPA** |  |  |  |  |  |  |  |  |  |  |  |  |  |
| Low (<600 METs･min/wk) | 112/1074 | 30.9 | 1.00 | (Ref.) |  |  | 1.00 | (Ref.) |  |  | 1.00 | (Ref.) |  |
| Moderate (600-3000 METs･min/wk) | 191/2800 | 19.7 | 0.66 | (0.52-0.83) | <0.001 |  | 0.68 | (0.54-0.86) | 0.002 |  | 0.69 | (0.54-0.87) | 0.002 |
| High (>3000 METs･min/wk) | 124/1742 | 20.7 | 0.72 | (0.56-0.94) | 0.013 |  | 0.76 | (0.58-0.98) | 0.038 |  | 0.77 | (0.59-1.00) | 0.050 |
|  | 427/5616 | 22.1 | Trend | | 0.026 |  | Trend | | 0.062 |  | Trend | | 0.069 |
| **ST** |  |  |  |  |  |  |  |  |  |  |  |  |  |
| <180 min/day | 67/1024 | 19.1 | 1.00 | (Ref.) |  |  | 1.00 | (Ref.) |  |  | 1.00 | (Ref.) |  |
| 180-299 min/day | 103/1409 | 21.2 | 1.02 | (0.75-1.38) | 0.916 |  | 0.98 | (0.72-1.33) | 0.877 |  | 0.97 | (0.71-1.32) | 0.821 |
| 300-479 min/day | 107/1558 | 19.9 | 0.90 | (0.66-1.22) | 0.501 |  | 0.86 | (0.63-1.18) | 0.353 |  | 0.86 | (0.63-1.17) | 0.327 |
| ≥480 min/day | 150/1625 | 26.9 | 1.16 | (0.87-1.55) | 0.306 |  | 1.12 | (0.83-1.50) | 0.459 |  | 1.09 | (0.81-1.46) | 0.580 |
|  | 427/5616 | 22.1 | Trend | | 0.317 |  | Trend | | 0.434 |  | Trend | | 0.509 |
| **Frail (n = 1583)** |  |  |  |  |  |  |  |  |  |  |  |  |  |
| **MVPA** |  |  |  |  |  |  |  |  |  |  |  |  |  |
| Low (<600 METs･min/wk) | 152/726 | 65.9 | 1.00 | (Ref.) |  |  | 1.00 | (Ref.) |  |  | 1.00 | (Ref.) |  |
| Moderate (600-3000 METs･min/wk) | 112/603 | 57.2 | 0.87 | (0.68-1.10) | 0.243 |  | 0.76 | (0.59-0.99) | 0.038 |  | 0.78 | (0.60-1.00) | 0.052 |
| High (>3000 METs･min/wk) | 29/254 | 34.1 | 0.52 | (0.35-0.78) | 0.001 |  | 0.48 | (0.32-0.71) | <0.001 |  | 0.49 | (0.32-0.73) | 0.001 |
|  | 293/1583 | 57.3 | Trend | | 0.002 |  | Trend | | <0.001 |  | Trend | | <0.001 |
| **ST** |  |  |  |  |  |  |  |  |  |  |  |  |  |
| <180 min/day | 41/244 | 51.7 | 1.00 | (Ref.) |  |  | 1.00 | (Ref.) |  |  | 1.00 | (Ref.) |  |
| 180-299 min/day | 45/323 | 42.4 | 0.77 | (0.51-1.18) | 0.234 |  | 0.72 | (0.46-1.11) | 0.135 |  | 0.72 | (0.47-1.12) | 0.143 |
| 300-479 min/day | 81/431 | 58.5 | 1.00 | (0.68-1.45) | 0.982 |  | 0.97 | (0.66-1.43) | 0.871 |  | 0.93 | (0.63-1.38) | 0.717 |
| ≥480 min/day | 126/585 | 67.1 | 1.14 | (0.80-1.62) | 0.469 |  | 1.08 | (0.75-1.57) | 0.670 |  | 1.03 | (0.71-1.50) | 0.872 |
|  | 293/1583 | 57.3 | Trend | | 0.118 |  | Trend | | 0.167 |  | Trend | | 0.302 |
| Model 1: Adjusted for baseline age and sex. | | | | | | | | | | | | | |
| Model 2: Adjusted for variables in Model 1 plus district, living situation, marital status, education, equivalent income, body mass index, hypertension, dyslipidemia, heart disease, stroke, diabetes mellitus, cancer, alcohol drinking status, smoking status, lower-back pain, knee pain, and frailty. | | | | | | | | | | | | | |
| Model 3: For MVPA, adjusted for variables in Model 2 plus ST. For ST, adjusted for variables in Model 2 plus MVPA. | | | | | | | | | | | | | |
| In stratified analyses for participants with/without frailty status, frailty was excluded from covariates. | | | | | | | | | | | | | |
| CI, confidence interval; FD, functional disability; HR, hazard ratio; METs, metabolic equivalents; MVPA, moderate-to-vigorous physical activity; PY = person-years; ST, sedentary time. | | | | | | | | | | | | | |

## Supplementary Figures

**Supplementary Figure 1.** Dose-response association between MVPA and FD, excluding FD that occurred during the first year of follow-up. Supplementary Figure 1A-1C shows the association between MVPA and FD among all participants (Supplementary Figure 1A), non-frail older adults (Supplementary Figure 1B), and frail older adults (Supplementary Figure 1C) after excluding individuals with incident FD during the first year of follow-up. MVPA and ST were mutually adjusted, as well as baseline age, sex, district, living situation, marital status, education, equivalent income, body mass index, hypertension, dyslipidemia, heart disease, stroke, diabetes mellitus, cancer, alcohol drinking status, smoking status, lower back pain, knee pain, and frailty (in the stratified analyses [Supplementary Figure 1B-1C], frailty was excluded from covariates). The reference value for MVPA was 0 METs▪min/week. Solid lines indicate hazard ratios for FD. Dashed lines indicate 95% confidence intervals. FD = functional disability; METs = metabolic equivalent; MVPA = moderate-to-vigorous physical activity; ST = sedentary time.

**Supplementary Figure 2.** Dose-response association between ST and FD, excluding FD that occurred during the first year of follow-up. Supplementary Figure 2A-2C shows the association between ST and FD among all participants (Supplementary Figure 2A), non-frail older adults (Supplementary Figure 2B), and frail older adults (Supplementary Figure 2C) after excluding individuals with incident FD during the first year of follow-up. ST and MVPA were mutually adjusted, as were baseline age, sex, district, living situation, marital status, education, equivalent income, body mass index, hypertension, dyslipidemia, heart disease, stroke, diabetes mellitus, cancer, alcohol drinking status, smoking status, lower back pain, knee pain, and frailty (in the stratified analyses [Supplementary Figure 2B-2C], frailty was excluded from covariates). The reference value for the ST was 300 min/day. Solid lines indicate hazard ratios for FD. Dashed lines indicate 95% confidence intervals. FD = functional disability; METs = metabolic equivalent; MVPA = moderate-to-vigorous physical activity; ST = sedentary time.
